# Medical Gossip

**Published:** 1870-05

**Authors:** 


***Medical Gossip.***

The Archbishop of Armagh sent to Cecil, Queen Elizabeth's Prime Minister, in 1571, this remedy for his gout: "Take two spaniel whelps of two days old, scald them and cause the entrails to be taken out, but wash them not. Take five ounces of brimstone, four ounces of turpentine, five ounces of parmaceti, a handful of nettles, and a quantity of oil of balm, and put all the aforesaid in them, stamped, and sew them up and roast them, and take the drops and anoint you where the grief is, and, by God's grace, your Honor shall find help." (*Froude*, *Hist. Eng.*, C. XXIV.) The Archbishop was otherwise known as Dr. Lancaster. Indeed, in the olden time, the functions of priest and doctor were often exercised by the same person, which is the probable explanation why it happens that doctors, now-a-days, are usually of a polemical turn, and clergymen love, above all things, to dabble in physic. ——About this same period, (1569,) the English soldiers sent to Ireland suffered grievously from flux, and it is curious to read that it was attributed to the continued eating of fresh beef—the Irish nation at that time living almost wholly on meat, and using but little bread. This was before the introduction of the potato. Throughout history, it is noticeable that all diseases are referred each to some single specific cause. The idea of concurrent causes is as essentially modern as it is correct.——When the spotted fever prevailed in Virginia, early in the '20's, Dr. Lucas, a practitioner, gave this treatment: (*Am. Med. Recorder*, Vol. V.) Evacuants very cautiously, always directing the patient to take a stiff drink of toddy after each stool. In many cases no sort of evacuation was premised or admissible. Patients took one or two ounces of Bark every one, two, or three hours, and from three to six pints of good rum in the 24 hours. Laudanum was given freely—many taking from eight to twelve drachms. Blood-letting, carried to the extent of producing an impression on the system, was almost uniformly fatal.

"Two stout, strong, athletic men took an emetic of tart. ant.; after puking moderately three times, they were prostrated to the lowest possible degree consistent with life. I found them without a pulse, in a profuse, cold, waxy sweat. The first took 60 drops of laud. in a wine-glass of strong rum, instanter; one ounce of eth. sulph., one of hartshorn, half a one of laud.; two ounces of bark and a pint of rum with 90 grains of camphor, were given him in about an hour; two ounces of bark, three of rum, one drachm of laud., and four of water, were directed to be administered every two hours by injection.

Large plasters of mustard were applied to the extremities, breast and spine, and equal parts of rum and a strong decoction of cyanne tea were given every half hour; this was continued for 48 hours, and in 72 hours he was free from danger. Boiling water was applied before the mustard, to ensure its effects. The other was treated in the same way, except boiling water; both are now well." A lady patient "took, every two hours, two ounces of bark, ten grs. of camphor, ten of vol. alkali, two scruples of laud., and three ounces of spirits, by injection, for 48 to 72 hours, and from 1 to 1½ pints of rum in the 24 hours." She recovered.

——In the same article he remarks that, "in a similar epidemic prevailing in 1819, 25 to 40 grs. of tart. ant. were required to produce vomiting — cathartics in proportion — and it was about impossible to salivate with mercury. "During this season, Thos. E. Abernathy took, in eight days, nine drachms of calomel, with a proportionable quantity of oil, salts and jalap, without an emetic, cathartic, or sialagogue effect. He recovered on the free use of bark, toddy, and elixir vitriol, and I never could tell what became of the calomel, etc. He had the disease in the dysenteric form." This will do for the sedative action of calomel — see Dr. LENTE. RIVERIUS, quoted by Bonet in 1682, urges, in what was probably the same disease, "blood-letting"— which "the change of type," perhaps, prevented Dr. Lucas from using. *Sic:*

"It is determined, by the wise judgment of Doctors, that when purple spots appear, in the beginning of the disease, and at those days when bleeding uses to be celebrated, if a sufficient quantity of bloud have not been taken away before, even at that time bloud may be taken away in a moderate quantity without any imminent danger; seeing that Eruption, which is in the beginning of the disease, is not critical but symptomatick, arising from the exceeding ebullition of the bloud and the ferment of malignant and putrefying humours: and therefore Nature's motion, which at that time is not, cannot be hindred: for if, when the body is plethorick, and sends out a thick and red urine, you do not let bloud on the score of spots appearing, Nature will scarce be able to conquer so great a quantity of humours, and there will be danger lest they fall upon some inner part, and breed in it a pernicious inflammation."

He also recommends, particularly, opening the Hæmorrhoidal veins by applying leeches to them, which latter seems to us, also, the most appropriate part on which to apply them.—— Henry C. Lea, the eminent medical book publisher, is achieving high reputation as a literary author. His books are quoted in Europe as among the most learned and able which this country has given to the world.——Prof. Richards has been recently delighting popular audiences in various parts of the Northwest by a series of brilliantly illustrated lectures on chemistry. Physicians can do much toward elevating popular taste by encouraging similar attempts to convey valuable information, whilst at the same time entertaining the people.—— The Foundling Hospital at New York has had over 150 babies left in its basket since last November.—— The *Pacific Journal* says there are eight institutions for the training of idiots in the United States. The largest is at Syracuse, N. Y., and has 150 pupils.—— The most important "vocative" now is the establishment of hospitals for inebriates, and such modification of the laws as will enable friends or the authorities to send the unfortunate victims to them, and to keep them there, *nolens volens.* —— The great specialist, RICORD, who has been on every side of every known professional question, has been officially appointed consulting physician to the Emperor Napoleon.—— Nelaton calls Napoleon's disease "vesical hæmorrhoids," since which valuable news was first announced, we have been consulted by at least a half-dozen old gonorrhœal veterans, who imagine they have the same disease.—— Senator Sumner failed to get the Senate to repeal the charter of the Med. Soc. of the District of Columbia. —— Jas. P. White, M. D., the retiring President of the N. Y. State Med. Soc., in his address, (*Buffalo Journal, March*, 1870,) chronicles the evidences of progress of medical science, and the catalogue should convince the most sceptical that the science is at anything except a stand-still.—— The Hospital question is still on the tapis in Chicago. The leading papers of the city advocate the views, first enunciated in this journal, as to the abandonment of the old castellated structures, though rich in fat contracts and stealings, and the adoption of the modern system. There should be ample spaces set apart in each division of the city for the purpose, cheap and numerous buildings put up, and, what has heretofore been neglected, an efficient ambulance corps organized. By the aid of the telegraph, ambulances could be ordered, when needed, as quickly as the fire engines. The present methods are simply barbarous.—— The *Richmond and Louisville Medical Journal* claims to be the largest medical monthly in the United States. —— The veteran Prof. JOHN W. DRAPER's miscellaneous works, philosophical and historical, are winning golden opinions and the most flattering notices, both in this country and Europe. As Metaphysicians always used to leave Physiology out of the question, so the Historians have been wont to omit all reference to those great physical influences which so certainly shape the character of individuals and nations. Prof. Draper's philosophical history of the recent civil war, with its causes and results, when contrasted with the *twaddle* which Mr. Froude uses to fill up the large interspaces of his occasionally brilliant descriptions, reflects honor upon the country, and the profession of Dr. Draper's choice. *L'esprit du corps* at least should make all doctors read his books. —— Six grains of Bicarb Pot. or Sod. added to each quart of fresh milk, before the cream has risen, bottled, tightly corked, and then placed in a water bath, heated to 190 degrees, and not more — the cork then coated with wax — is the Parisian method of preserving milk for a long period.—— The fancy envelopes, green on the inside and white without, are poisonous from arsenite of copper in the coloring matter. *Cave!*——A "Society for the Suppression of Useless Information" is announced.—— Among the most remarkable instances of *ante mortem* beneficence, we see announced that Drs. C. L. Ives and A. S. Munson have given $1,000 each to Yale Medical College.—— Another case of "*Railway Spine*" has been before the Chicago courts. A remarkable peculiarity in this case was the entire absence of those symptoms supposed to be diagnostic of spinal meningitis or myelitis. No febrile symptoms, disorder of respiratory, digestive or defecatory organs, no paralysis of sensation or motion, loss of weight, or physiognomic change. Chicago hopes, with this case, to keep up with New York and its McFarland variety of insanity.—— Ninety to one hundred deaths every week from epidemic small pox in Paris.—— At the University of Zurich it is said the lady students are treated with great courtesy and deference by the males. A Miss Morgan recently graduated with distinction in the departments of Medicine, Surgery and Midwifery.—— A Miss Garrett has lately been appointed one of the physicians of the East London Hospital for children. "The cry is, still they come," (not the children, but the doctresses).—— Speaking of women and children, a case of *ovarian pregnancy* has recently occurred in this city. Death resulted near the third month, rupture of the ovarian cyst occurring with fatal hœmorrhage into the abdominal cavity. We saw the specimen some months since, and were promised a full report, with appropriate illustrations, but from some unaccountable reason we have not yet received the manuscript.—— A few days since, we attended a case of labor, "short, sharp, and decisive," in which occurred rupture of the trachea, during violent straining efforts, with resulting emphysema of the neck and upper part of the thorax. At one time this was so considerable as to interfere somewhat with respiration, and produce lividity of the face, from obstruction to the return circulation. The trouble is spontaneously abating. We dislike to say much about such cases as these last two, for fear the sex will quit the business of bearing children, and turn doctors, (or doctresses — which is it?) —— To avoid the disastrous tendency toward keeping down excess of population, so much complained of, it is well to recollect that concentrated infusion of digitalis, according to Dr. Besnier, applied in saturated compresses according to the patient's preferences, has been found an encouraging practice in orchitis. Double orchitis very often if not always, endangers subsequent obedience to the commandment, "Multiply," etc.——The question of the possible duration of pregnancy has lately been raised before a British court. Sir James Simpson "qualified" to a case of 310 days. In the case before court, intercourse 301 days before the birth gave the plaintiff £200 damages. Served him right.—— One of our contemporaries objects to the term LOOT, which we affix to the section of the JOURNAL containing valuable plunder from all sources. Clearly it is not "up" in oriental literature. We certainly shall not seek our *loot* from the columns of the secular papers, or "chronicle small beer," practices we might possibly charge upon "one of our contemporaries," were we anxious to say, "You're another!" It is all a matter of taste.—— Phosphorus is recommended in the treatment of psoriasis. It is to be exhibited both internally and externally.—— Moûtre uses for prurigo, suppositors composed of Iodoform and cocoa butter: ℞ Iodoform, gr. xx; Butyr. Cacao. ʒj. M. In suppositors vj. divid. For frictions use a drachm of the Iodoform to the ounce of simple ointment.—— The operation of transfusion is getting frequent. After defibrination of the blood, as practiced by Prof. Freer, the operation becomes almost as si mpleas the hypodermic injection, and nearly devoid of danger. No complicated apparatus is required. Nothing but reasonable care on the part of the operator.

